# Evaluation of autoantibodies and immunoglobulin G subclasses in women with suspected macroprolactinemia

**DOI:** 10.1002/jcla.23456

**Published:** 2020-06-29

**Authors:** Chao Yu, Fei Fan, Siqi Hu, Lingxin Meng, Dong Xu, Juan Wang, Lu Chen, Jingrui Liu, Ying Dong, Yifan Lu, Min Shen, Yanhong Zhai, Zheng Cao

**Affiliations:** ^1^ Department of Laboratory Medicine Beijing Obstetrics and Gynecology Hospital Capital Medical University Beijing China; ^2^ Institute of Pathogen Biology, and Center for AIDS Research Chinese Academy of Medical Sciences & Peking Union Medical College Beijing China; ^3^ Department of Gynecological Endocrinology Beijing Obstetrics and Gynecology Hospital Capital Medical University Beijing China; ^4^ Reference Laboratory MedicalSystem Biotechnology Co., Ltd Ningbo China

**Keywords:** autoantibody, IgG subclass, macroprolactinemia, PEG, prolactin

## Abstract

**Background:**

Macroprolactin mostly composed of an immunoglobulin G (IgG) and a monomeric prolactin (PRL) represents the major circulating PRL form in the patients with macroprolactinemia that are usually asymptomatic and may not require treatment. In this study, we aimed to evaluate the prevalence of antithyroid and antinuclear antibodies, as well as the IgG subclass distributions in the patients suspected for macroprolactinemia.

**Methods:**

From January to July in 2018, totally 317 patients with elevated PRL were subjected to the polyethylene glycol (PEG) precipitation assay. The patients with recovery rates of ≤60% were subjected for IgG subclass determination and autoantibody testing including thyroid peroxidase antibody (aTPO), antithyroglobulin antibody (aTG), and antinuclear antibodies (ANA).

**Results:**

The higher the post‐PEG PRL recovery rates, the less typical hyperprolactinemia symptoms and the higher prevalence of autoantibodies were observed. The IgG1 and IgG3 were the predominant subclasses in the PRL‐IgG complexes according to the immunoprecipitation experiments.

**Conclusion:**

The patients with post‐PEG PRL recovery rates of <40% and 40%‐60% were likely to represent two distinct populations of different clinical presentations. The prevalence of autoantibodies and IgG subclasses distribution suggested their pathogenic significance in the development of macroprolactinemia.

## Graphical abstract

To evaluate the prevalence of associated autoantibodies, as well as the IgG subclass distributions in the patients suspected for macroprolactinemia, totally 317 patients with elevated prolactin were recruited for the initial screening with the polyethylene glycol precipitation assay. The patients with recovery rates of ≤60% were further subjected to IgG subclass determination and autoantibody testing including thyroid peroxidase antibody, antithyroglobulin antibody, and antinuclear antibodies, followed by proper statistical analysis.

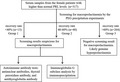

## 1 INTRODUCTION

Hyperprolactinemia is a physiologic or pathologic condition that causes hypersecretion of prolactin (PRL) by lactotroph cells.^1^ It was thought to be present in 10%‐25% women with secondary amenorrhea or oligomenorrhea, in approximately 30% of women with galactorrhea or infertility, and in 75% of those with both amenorrhea and galactorrhea.^2^, ^3^, ^4^

In human serum, three main species of PRL have been identified including the monomeric PRL (molecular mass 23 kDa) being the predominant form, the big PRL (molecular mass 50‐60 kDa) and the big‐big or macroprolactin (molecular mass 150‐170 kDa).^5^ Jackson et al first used the new term “macroprolactinemia” to describe a patient with marked hyperprolactinemia whose PRL mainly consisted of macroprolactin.^6^ In the great majority of cases, macroprolactin was composed of a complex formed by an immunoglobulin G (IgG) and a monomeric PRL.^7^, ^8^ Furthermore, in rare cases with slightly elevated PRL levels, non–IgG‐bound forms of macroprolactin including complexes with IgA or IgM, highly glycosylated monomeric PRL, covalent, or noncovalent aggregates of monomeric PRL, have also been demonstrated.^9^

The gold standard technique for the diagnosis of macroprolactinemia is the gel filtration chromatography (GFC), which is accurate, reproducible but also expensive, time‐consuming, and labor intensive.^10^ The polyethylene glycol (PEG) precipitation has been widely used as a screening method, with which a number of studies showed that the PEG‐induced precipitation of macroprolactin in serum sample represented a simple, inexpensive, and reliable screening assay for hyperprolactinemia differentiation.^5^, ^11^ This test enabled the correct diagnosis of macroprolactinemia in at least 80% of the cases.^12^, ^13^, ^14^

Macroprolactinemia is mostly defined as a type of hyperprolactinemia where more than 60% of circulating PRL is made up of macroprolactin.^12^, ^13^, ^14^, ^15^ The recovery of PRL of >60% after precipitation with PEG 6000 usually indicated that macroprolactin was not present in significant amounts.^16^ With the recovery rate of 40%‐60%, macroprolactin might be present and the lower the recovery the less likely this was the case of true hyperprolactinemia; with the recovery rate of <40%, it was typically consistent with the presence of substantial quantities of macroprolactin.^13^, ^14^, ^15^, ^17^

As the native state of macroprolactin is confined to the intravascular space, macroprolactin was shown to have differing degrees of in vitro biologic activity in most studies^9^, ^18^ and it was found that most macroprolactinemic patients were asymptomatic.^9^ Several studies have identified the anti‐PRL autoantibodies in the sera of patients with macroprolactinemia,^19^, ^20^, ^21^, ^22^, ^23^ consistent with the fact that most of the macroprolactinemia cases possessed PRL‐IgG complexes.^24^

Macroprolactinemia was considered to be a common finding in endocrinological practice with relative high incidence rates in the hyperprolactinemia population.^10^, ^25^, ^26^ Despite of many reports about the prevalence, laboratory diagnosis, and clinical manifestations of macroprolactinemia,^1^, ^5^, ^14^, ^27^, ^28^ little is known about its causes and the nature of the antibodies associated with the PRL‐IgG complexes. In the present study, we aimed to evaluate the prevalence of antinuclear antibodies (ANA) and antithyroid antibodies in the patients with suspected macroprolactinemia. Moreover, the IgG subclasses of the PRL‐IgG complexes were investigated and compared between the patient groups with different post‐PEG recovery rates.

## 2 MATERIALS AND METHODS

### 2.1 Patients

From January to July in 2018, 40 061 female patients visiting the Endocrinology Department of the Beijing Obstetrics and Gynecology Hospital were tested for the serum PRL levels. The subjects (between 20 and 40 years old) with prolactin >30 ng/mL (the upper limit of the current prolactin reference interval used in the laboratory) were included in the present study, in combination of the following exclusion criteria: pregnancy and lactation, under certain medication such as anti‐depressant drugs, anti‐hypertensive drugs, anti‐gastric acid drugs, and some medical conditions other than pituitary tumors causing PRL abnormalities such hypothyroidism.

This study was approved by the Ethics Committee of Beijing Obstetrics and Gynecology Hospital. Two milliliter venous blood was collected from each of the recruited patients followed by centrifugation and serum separation.

### 2.2 Reagents and methods

The serum prolactin was determined by the Siemens Centaur XP Chemiluminescent Immunoassay platform (Siemens, Ireland) with the prolactin reagent kit (Siemens, Cat. No. 09505871, USA). The macroprolactin screening was performed by the polyethylene glycol (PEG) 6000 (Sigma‐Aldrich, Cat. No. 8074911000, Germany) experiment as previously described.^29^ Briefly, 200 μL of the PRL‐elevated serum was well mixed with 200 μL of 25% PEG, followed by centrifugation 1500 × *g* for 30 minutes. The supernatant was re‐analyzed for PRL, and the PRL recovery was calculated with the following equation: (2 × PRL level following PEG treatment/PRL level before PEG treatment) × 100%.^29^

The thyroid peroxidase antibody (aTPO) IgG (Siemens, Cat. No. 10630887, USA) and the antithyroglobulin antibody (aTG) IgG (Siemens, Cat. No. 10492399, USA) were also measured by the Siemens Centaur XP instrument mentioned above. The assay for antinuclear antibodies (ANA) was performed by the ELISA method on the TECAN Freedom EVOlyzer^®^ (Switzerland) platform, with the ANA detection kits obtained from AESKU Diagnostics (Cat. No. 3119, Germany). The ELISA experiments were performed according to the manufacturer's instructions. Briefly, the diluted sera were incubated in 96‐well microplates for 30 minutes at room temperature. After the washing step, the conjugate was incubated and washed again before adding the substrate to generate enzymatic colorimetric reactions. The concentration of target antibody was calculated based on its OD (at the wavelength of 450 nm) value compared with the standard curve.^30^

### 2.3 Measurement of IgG subclasses of anti‐PRL autoantibodies

To evaluate the subclasses of IgG bound to the serum PRL, an immunoprecipitation experiments were carried out. Briefly 100 μL of each serum sample was incubated with the prolactin monoclonal antibody (Thermofisher, MIP0202, USA) cross‐linked agarose (Enriching, MAg25K/NHS kit, China) at 4°C overnight with continuous shaking. After washing three times with PBS, the bound anti‐PRL antibody‐PRL‐IgG complexes were then eluted with 0.1 M sodium citrate (pH 3.0) and further assayed by Western blotting. The Western blotting was performed as previously described.^31^ The eluted complexes of interest were separated in the SDS‐12% PAGE (Beijing Biotides Biotechnology, WB1103, China) and transferred onto the nitrocellulose membranes (Whatmann). The membranes were then probed with the anti‐human IgG1 antibody (ThermoFisher, A10648, USA), anti‐human IgG2 antibody (SouthernBiotech, 9070‐01, USA), anti‐human IgG3 antibody (SouthernBiotech, 9210‐01), or anti‐human IgG4 antibody (SouthernBiotech, 9200‐01) separately, followed by incubation with the IRDye™ secondary antibodies (1:20 000). Along with the patient serum, 0.5 ug of pure IgG1 (SouthernBiotech, 0151L‐01, USA), IgG2 (Bio‐Rad, 5225‐3004, USA), IgG3 (SouthernBiotech, 0153L‐01, USA), or IgG4 (Sigma‐Aldrich, I4764, Germany) was loaded in each SDS‐PAGE. The protein bands were visualized on a LiCor Odyssey instrument (LI‐COR Biosciences, USA). The intensities of protein bands (IgG1‐IgG4) in Western blots were determined with the ImageJ software (National Institutes of Health, USA) and normalized against the pure IgG1‐IgG4 proteins.

### 2.4 Statistical analysis

The statistical analyses were performed using the SPSS software version 21.0 (IBM, USA). The differences between groups were compared by nonparametric Mann‐Whitney *U* test. Categorical variables were compared using the chi‐square test with Yates's correction. A *P* value of <.05 was considered as statistically significant.

## 3 RESULTS

### 3.1 Clinical presentations in the patients with suspected macroprolactinemia

**FIGURE 1:**
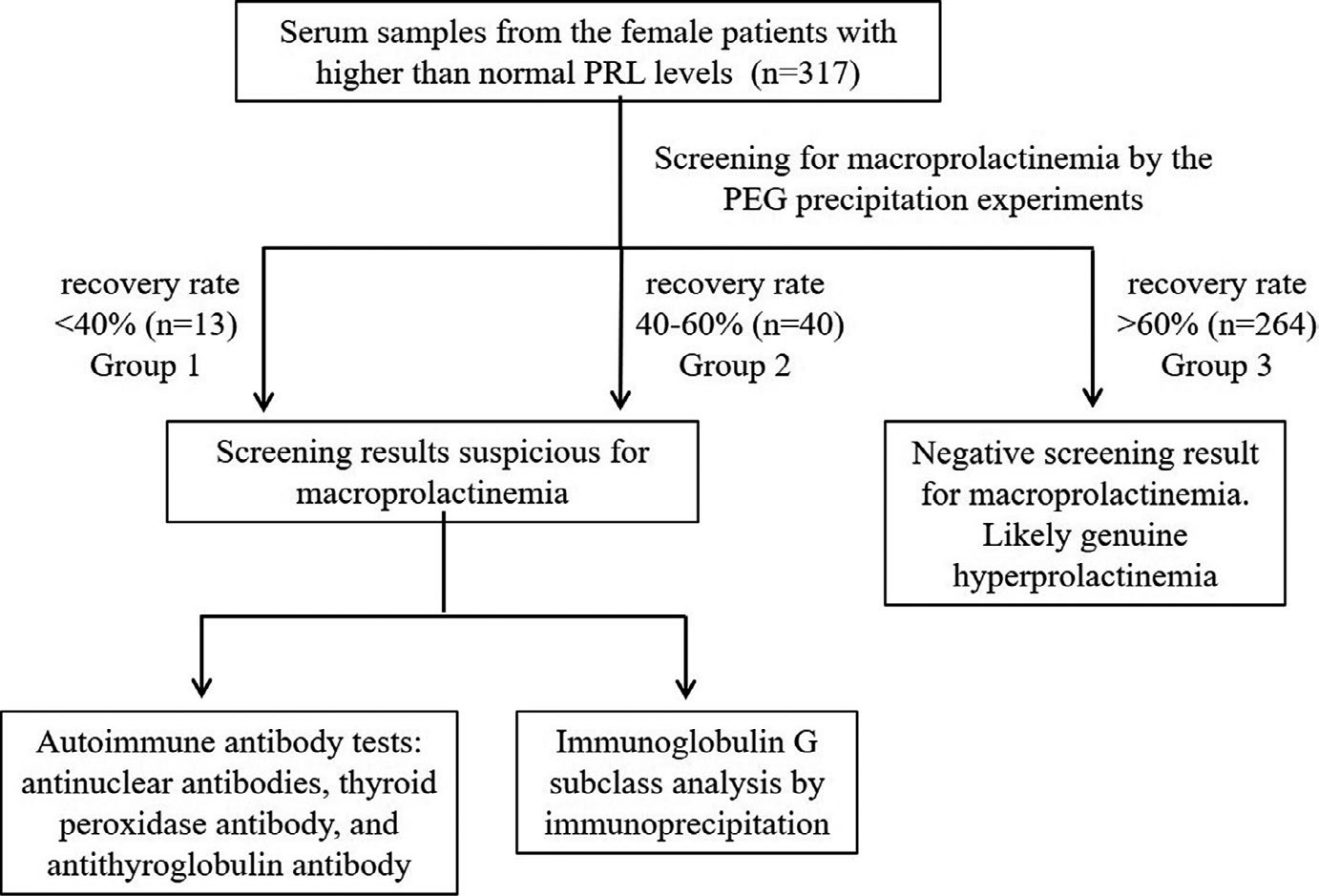
Schematic diagram for patient recruitment and study design

Of the 40 061 women visiting the Endocrinology Department, totally 317 patients with elevated serum prolactin level and meeting the exclusion criteria were subsequently subjected to the PEG precipitation screening assay. As shown in Figure 1, with the PEG screening, only 13 subjects had a PRL recovery rate of <40% (Group 1), compared with the 40 subjects with a recovery rate of 40%‐60% (Group 2). As expected, the majority of the enrolled patients (n = 264) showed a recovery rate of >60% (Group 3), indicating the group of “true hyperprolactinemia.” More interestingly, the percentage of the patients with the typical clinical presentations (including decreased libido, infertility, gynecomastia, decreased bone mass, and galactorrhea) of the hyperprolactinemia in Group 1 (23.0%) was significantly lower than that in Group 2 (67.5%) and Group 3 (80.7%). In other words, the relative amount of the macroprolactin was negatively associated with the prevalence of the classic symptoms of the true hyperprolactinemia (Table 1).

**TABLE 1:** Associations between typical hyperprolactinemia symptoms and PEG recovery rate

	<40% recovery Group 1 (n = 13)	40%‐60% recovery Group 2 (n = 40)	>60% recovery Group 3 (n = 264)
% of typical hyperprolactinemia symptoms^a^ (number/total)	23.0% (3/13)	67.5% (27/40)	80.7% (213/264)

	Group 1 vs 2	Group 2 vs 3	Group 1 vs 3
*P* value (chi‐square test)	.003	.090	<.001

^a^ Decreased libido, infertility, gynecomastia, decreased bone mass, and galactorrhea.

### 3.2 Associations between autoantibodies and PRL recovery rates

As autoimmunity has been related to the prevalence and pathogenesis of hyperprolactinemia,^32^ the following autoantibodies including ANA, aTPO, and aTG were tested for all the Group 1 and Group 2 patients that were suspected for macroprolactinemia with low PEG recovery rates (<60%) (Figure 1). For control purpose, a portion of randomly selected Group 3 patients were also tested for ANA (n = 98) and antithyroid autoantibodies (n = 10) (due to limited serum accessibility). As summarized in Table 2, the higher incidence rates of ANA, aTPO, and aTG were associated with the greater PRL recovery rates post‐PEG precipitation, although the differences between Group 1 (46.1% for ANA, 7.7% for aTPO or aTG) and Group 2 (70.0% for ANA, 12.5% for aTPO or aTG) were not statistically significant. Interestingly, with the comparison between Group1 and Group 3 in Table 2, no statistical difference was found for the positive rates of ANA or antithyroid autoantibodies (aTOP and aTg), suggesting that non–PRL‐specific autoantibodies did not significantly contribute to the PEG precipitation. For the ANA testing, with close positive rates for Group 1 (46.1%) and Group 3 (41.8%), a significant difference was only observed between Groups 2 and 3 (*P* = .003) but not between Groups 2 and 1 (*P* = .221), most likely due to the statistical power difference introduced by different sample sizes of Groups 1 (n = 13) and 3 (n = 98) (Table 2).

**TABLE 2:** Associations between autoantibody positivity and PEG recovery rates

	% of ANA^a^ positivity	% of aTPO^b^ or aTG^c^ positivity
Group 1 (n = 13)	46.1% (6/13)	7.7% (1/13)
Group 2 (n = 40)	70.0% (28/40)	12.5% (5/40)
Group 3 (n = 98)	41.8% (41/98)	10.0% (1/10)^e^
*P* value^d^ (Group 1 vs 3)	.767	.848
*P* value (Group 2 vs 3)	.003	1.000
*P* value (Group 1 vs 2)	.221	.977

^a^ Antinuclear antibodies.

^b^ thyroid peroxidase antibody.

^c^ antithyroglobulin antibody.

^d^ *P* value calculated from chi‐square test.

^e^ 10 random serum samples were tested from Group 3 for aTPO and aTG.

### 3.3 IgG Subclasses of anti‐PRL autoantibodies

**FIGURE 2:**
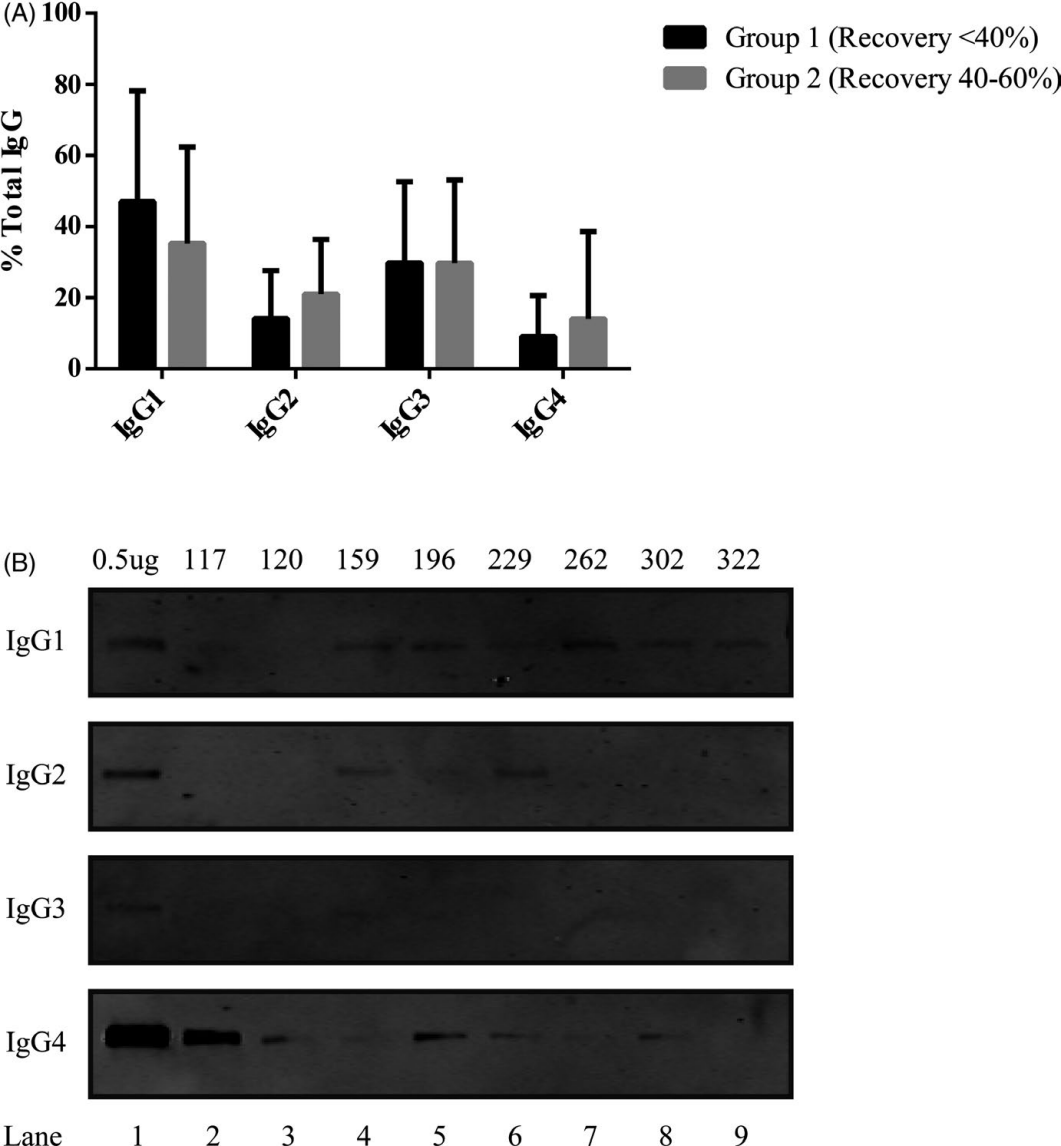
Immunoprecipitation with the serum samples of the patients suspected for macroprolactinemia. A, Relative levels of PRL‐specific IgG subclasses associated with PRL‐IgG complexes in patients with anti‐PRL autoantibodies are shown in bar graph. Data are expressed as mean ± standard deviation. B, Representative Western blots with the patients’ serum samples from Group 1 (#117, 159, 196, 229) and Group 2 (#120, 262, 302, 322). Lane 1: positive control loaded with pure IgG1‐IgG4 proteins

In the immunoprecipitation assay, the serum PRL was first trapped to the anti‐human PRL antiserum‐coated beads; then, the IgG subclasses from the PRL‐IgG complexes were determined using mouse anti‐human IgG subclass‐specific antibody. All Group 1 and 2 patients were tested positive in the immunoprecipitation assay for anti‐PRL IgG subclass determination. After normalizing to the pure commercial IgG subclass‐specific proteins loaded along with the patient samples onto the SDS‐PAGE, the average IgG subclass percentages of total IgG (sum of relative quantities of IgG1, IgG2, IgG3, and IgG4) in Group 1 were determined as follows (in the decreasing order): 47.0% for IgG1, 29.8% for IgG3, 14.1% for IgG2, and 9.1% for IgG4 (Table 3, Figure 2). Interestingly, the same IgG subclass ranking was seen with the Group 2 patients: 35.3% for IgG1, 29.7% for IgG3, 21.0% for IgG2, and 14.0% for IgG4 (Table 3, Figure 2). With the Mann‐Whitney *U* test, the relative levels of IgG1 were significantly higher than those of IgG2 and IgG4 in both Group 1 and Group 2. Similarly, the relative levels of IgG3 were significantly higher than those of IgG 4 in both Groups 1 and 2. However, the statistically significant difference between IgG2 and IgG4 was only found in Group 2, suggesting the heterogenicity of the anti‐PRL antibodies in the populations with different post‐PEG recovery rates (Table 3).

**TABLE 3:** IgG subclass distributions in Group 1 and Group 2 patients

Average of % total IgG	IgG1	IgG2	IgG3	IgG4		
Group 1^a^	47.0%	14.1%	29.8%	9.1%		
Group 2^b^	35.3%	21.0%	29.7%	14.0%		

*P* values Mann‐Whitney *U* test	IgG1 vs IgG2	IgG1 vs IgG3	IgG1 vs IgG4	IgG2 vs IgG3	IgG2 vs IgG4	IgG3 vs IgG4
Group 1	0.002	0.340	0.002	0.140	0.213	0.026
Group 2	0.045	0.363	<0.001	0.217	0.006	0.001

^a^ 40% recovery after PEG precipitation.

^b^ 40%‐60% recovery after PEG precipitation.

## 4 DISCUSSION

The study was designed to investigate the laboratory and clinical significance of the women suspected for macroprolactinemia due to decreased PRL recover rates post‐PEG precipitation. As the macroprolactinemic patients with significant amount of prolactin‐IgG complexes are less likely to exhibit the classic symptoms of the hyperprolactinemic syndrome,^33^ it is therefore important to distinguish such individuals from those with true hyperprolactinemia to avoid unnecessary biochemical and imaging investigations or even inappropriate medical treatment.^5^, ^34^, ^35^

With the PEG precipitation screening assay that was universally adopted by clinical laboratories, 4.1% (13/317) of the enrolled patients with elevated serum PRL had the recovery rates of <40%, and 12.6% (40/317) had the recovery rates of 40%‐60% (Figure 1), which was close to other findings.^33^, ^36^ Many previous reports have indicated that the post‐PEG recovery rate of 40% was an acceptable cutoff for macroprolactinemia screening purpose.^15^, ^16^, ^17^, ^37^, ^38^, ^39^ However, because the PRL antibodies used in the commercial kits have different antigen specificity and reactivity,^40^, ^41^, ^42^ the incidence rates of suspected macroprolactinemia were highly variable, between 15% and 35%.^10^, ^25^, ^26^, ^43^, ^44^ As a result, Chen et al re‐evaluated the cutoff of the recovery rates for the PEG screening assay and found that 50% for the i2000sr (Abbott Laboratories) and 60% for the E170 (Roche Diagnostics) were optimum thresholds that were further verified by the GFC method.^40^ In our study, with the platform of Siemens Centaur XP used for PRL measurement, the patients of Group 1 (<40% recovery) and Group 2 (40%‐60% recovery) seemed to be two distinct populations with variable manifestations of classic hyperprolactinemia symptoms (Table 1). Therefore, whether a different post‐PEG recovery rate cutoff other than 40% exits in our PRL testing system needs to be further verified in combination with the gold standard GFC method.

It has been shown that autoimmune disorders were accompanied by increased PRL levels.^45^, ^46^ Pelkonen et.al reported three hyperprolactinemia cases in a 12 euthyroid‐patient cohort.^47^ In another study, PRL was found to be significantly elevated in the patients with Hashimoto's thyroiditis which is introduced by autoantibodies targeting thyroid.^48^ Similarly, Kramer CK, et al observed increased prevalence of antithyroid antibodies in the presence of genuine hyperprolactinemia or macroprolactinemia, evidencing the association of PRL increase and antithyroid autoimmunity.^49^

The hyperprolactinemia has been reported in the patients with different autoimmune disorders, such as systemic lupus erythematosus (SLE), antiphospholipid syndrome, rheumatoid arthritis (RA), and a spectrum of connective tissue diseases.^32^ The ANA are a group of autoantibodies that bind to contents of the cell nucleus, and the test is widely used as an indicator for most of the autoimmune disorders mentioned above. The lower prevalence of both ANA and antithyroid antibodies in Group 1 than Group 2 supported the idea that non‐specific autoantibodies such as ANA or antithyroid antibodies could precipitate with PEG less efficiently than the PRL‐specific antibody.

Little is known about the mechanisms involved in the development of anti‐PRL autoantibodies, although it was hypothesized that certain genetic background might confer extra susceptibility to such condition.^32^ On the other hand, it was proposed that determination of IgG subclasses of anti‐PRL autoantibodies might be helpful to elucidate their pathogenic significance, as IgG autoantibody subclasses were reported to have different biological properties.^50^ In a study with a smaller group of macroprolactinemia patients (n = 6) reported by Hattori et al, it was found that IgG4 was the major subclass as it was observed in five of the six patients included, suggesting chronic antigen stimulation in those patients. With the similar experimental strategy to trap and determine the IgG subclasses of the PRL‐IgG complexes but larger population suspected for macroprolactinemia (13 patients in Group 1 and 40 patients in Group 2), we found that IgG1 and IgG3 were the predominant IgG species in both Groups 1 and 2. Interestingly, in general IgG1 and IgG3 were more likely found in nonorgan‐specific autoimmune conditions such as SLE and RA.^50^ Therefore, the origin and the development of the anti‐PRL autoantibodies might share some similarity with those identified in SLE and RA.

In conclusion, a significant portion (53/317) of the patients with elevated PRL were suspected for macroprolactinemia with the PEG precipitation screening. The patients with post‐PEG PRL recovery rates of < 40% (Group 1) and 40%‐60% (Group 2) were likely to represent two distinct populations of different clinical presentations, although the PRL assay‐specific post‐PEG recovery cutoff needs to be further optimized in our testing system. Lastly, the IgG1 and IgG3 were the predominant subclasses in the PRL‐IgG complex trapped by the immunoprecipitation method, suggesting their pathogenic significance in the development of anti‐PRL autoantibodies.

## CONFLICT OF INTEREST

The authors declare no conflict of interest. The sponsor had no role in the design, execution, interpretation, or writing of the study.
